# Noise-invariant Neurons in the Avian Auditory Cortex: Hearing the Song in Noise

**DOI:** 10.1371/journal.pcbi.1002942

**Published:** 2013-03-07

**Authors:** R. Channing Moore, Tyler Lee, Frédéric E. Theunissen

**Affiliations:** 1 Biophysics Graduate Group, University of California, Berkeley, Berkeley, California, United States of America; 2 Helen Wills Neuroscience Institute, University of California, Berkeley, Berkeley, California, United States of America; 3 Department of Psychology, University of California, Berkeley, Berkeley, California, United States of America; Northwestern University, United States of America

## Abstract

Given the extraordinary ability of humans and animals to recognize communication signals over a background of noise, describing noise invariant neural responses is critical not only to pinpoint the brain regions that are mediating our robust perceptions but also to understand the neural computations that are performing these tasks and the underlying circuitry. Although invariant neural responses, such as rotation-invariant face cells, are well described in the visual system, high-level auditory neurons that can represent the same behaviorally relevant signal in a range of listening conditions have yet to be discovered. Here we found neurons in a secondary area of the avian auditory cortex that exhibit noise-invariant responses in the sense that they responded with similar spike patterns to song stimuli presented in silence and over a background of naturalistic noise. By characterizing the neurons' tuning in terms of their responses to modulations in the temporal and spectral envelope of the sound, we then show that noise invariance is partly achieved by selectively responding to long sounds with sharp spectral structure. Finally, to demonstrate that such computations could explain noise invariance, we designed a biologically inspired noise-filtering algorithm that can be used to separate song or speech from noise. This novel noise-filtering method performs as well as other state-of-the-art de-noising algorithms and could be used in clinical or consumer oriented applications. Our biologically inspired model also shows how high-level noise-invariant responses could be created from neural responses typically found in primary auditory cortex.

## Author Summary

Birds and humans excel at the task of detecting important sounds, such as song and speech, in difficult listening environments such as in a large bird colony or in a crowded bar. How our brains achieve such a feat remains a mystery to both neuroscientists and audio engineers. In our research, we found a population of neurons in the brain of songbirds that are able to extract a song signal from a background of noise. We explain how the neurons are able to perform this task and show how a biologically inspired algorithm could outperform the best noise-reduction methods proposed by engineers.

## Introduction

Invariant neural representations of behaviorally relevant objects are a hallmark of high-level sensory regions and are interpreted as the outcome of a series of computations that would allow us to recognize and categorize objects in *real life* situations. For example, view-invariant face neurons have been found in the inferior temporal cortex [1] and are thought to reflect our abilities to recognize the same face from different orientations and scales. The representation of auditory objects by the auditory system is less well understood although neurons in high-level auditory areas can be very selective for complex sounds and, in particular, communication signals[2]. It has also been shown that auditory neurons can be sound level invariant [3], [4] or pitch sensitive [5]. As is the case for all neurons labeled as invariant, pitch sensitive neurons respond similarly to many different stimuli as long as these sounds yield the same pitch percept. Both sound level invariant and pitch sensitive neurons could therefore be building blocks in the computations required to produce invariant responses to particular auditory signals subject to distortions due to propagations or corruption by other auditory signals. The existence of such distortion invariant auditory neurons, however, remains unknown. Similarly, the neuronal computations required to recognize communication signals embedded in noise are not well understood although it is known that humans [6] and other animals [7] excel at this task.

In this study, we examined how neurons in the secondary avian auditory cortical area NCM (*CaudoMedial Nidopalium*) responded to song signals embedded in background noise to test whether this region presents noise-invariant characteristics that could be involved in robust song recognition. We chose the avian model system because birds excel at recognizing individuals based on their communication calls [8], often in very difficult situations [9]. Moreover, the avian auditory system is well characterized and it is known that neurons in higher-level auditory regions can respond selectively to particular conspecific songs [10]. We focused our study on NCM because a series of neurophysiological [11], [12] and immediate early gene studies [13], [14] have implicated this secondary auditory area in the recognition of familiar songs. In addition, although neuronal responses in the primary avian auditory cortex regions are systematically degraded by noise [15], studies using immediate early gene activation suggested that responses to conspecific song in NCM were relatively constant for a range of behaviorally relevant noise levels [16].

## Results/Discussion

We recorded neural responses from single neurons in NCM of anesthetized adult male Zebra Finches. We obtained responses to 40 different unfamiliar conspecific songs and to the same songs embedded in naturalistic synthetic noise also called modulation-limited noise (ml-noise from here on). Ml-noise is broadband white-noise that has been filtered in the modulation domain to mimic the structure that is found in environmental sounds by restricting the power of modulations in the envelope to low spectral-temporal frequencies [17]. Ml-noise has also been shown to be an efficient stimulus for driving high-level auditory neurons (see Methods for additional details). The signal to noise ratio (SNR) was set at 3dB.

### Noise Invariant Neurons in NCM

**Figure 1:**
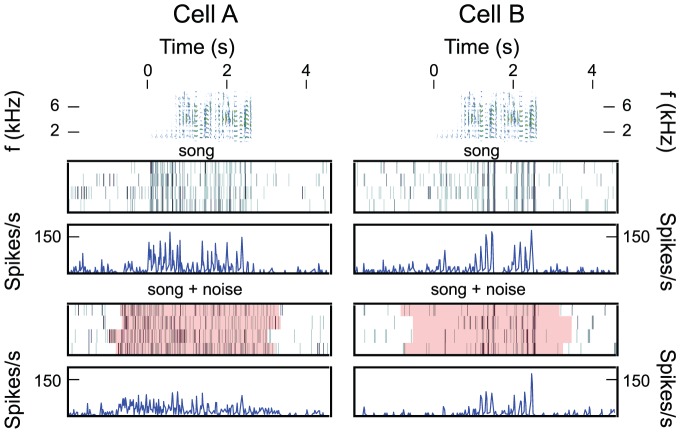
Noise-invariant responses in the avian NCM. Responses of two neurons (Cell A and Cell B) to song presented alone and over noise. The top row shows the spectrogram of the same zebra finch song used in the two recordings. Song starts at 0s. Below the spectrogram are raster plots and corresponding smoothed PSTHs. The first raster and PSTH correspond to the response of each neuron to the song alone presented at 70 dB SPL. Clear temporal synchrony across the four trials can be seen illustrative of an equally robust response to song stimuli. The second raster and PSTH correspond to the responses to song+ modulation limited noise (ml-noise) presented at 3dB signal to noise ratio. Ml-noise is synthesized by low-pass filtering white noise in the space of temporal and spectral modulations (see methods). The pink highlights show the duration of the stimulus (song + noise). The onset and offset of the stimulus is different in each trial because the trials are aligned to the onset of the song and the noise masker began and ended with a different delay in each trial. The noise was also different in each trial. This addition of naturalistic noise destroys the cross-trial synchrony in the response for the neuron shown in the left column but not for the neuron shown in the right column.

As illustrated on the left panels in Fig. 1, responses of some neurons to song signal were almost completely masked by the addition of noise. In these situations, the post-stimulus time histogram (PSTH) obtained for song only (third row) is very different than the one obtained for song + ml-noise (fifth row). However, some neurons also showed strong robustness to noise degradation as illustrated on the right panels of Fig. 1. Those neurons had similar PSTHs for both conditions.

To quantify the degree of noise robustness, we calculated two measures of noise-invariance: a de-biased correlation coefficient between the PSTHs obtained for the song alone and song + ml-noise stimuli (called I_CC_) and the ratio of the SNR estimated for the song + noise response and the song + ml-noise response (I_SNR_ invariance). The I_CC_ metric is a normalized measure that ranges in values between −1 and 1. It is 1 when the response pattern observed to song+ml-noise is identical to the one observed to song, irrespective of the relative magnitude of the two responses. For I_SNR_, we defined the response SNR as follows. For the response to song alone, the signal power was defined as the variance in the PSTH across time and the noise was defined as the mean firing rate. For the response to song plus noise, the signal was taken to be the time-varying response that could be predicted linearly from the response to song alone and the noise was the mean of this predicted response (see methods). This second value of invariance is bounded between 0 and 1 and captures not only the similarities in response patterns but also magnitudes of time-varying responses that carry information about the song. As shown in the supplemental material, the two measures were highly correlated and subsequent analyses resulted in very similar results and identical conclusions. For brevity, we show the analysis using the I_CC_ metric in the main paper. Some of the results with the I_SNR_ metric are included in the supplemental material.

### Noise Invariance and Frequency Tuning

**Figure 2:**
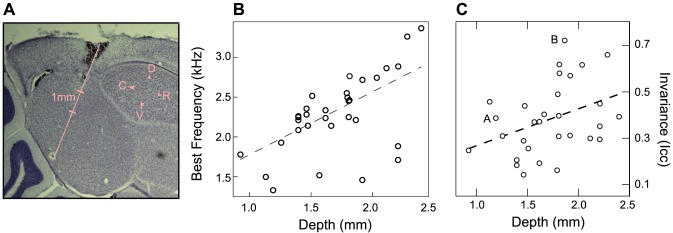
Location of noise invariant neurons in NCM. **A.** Photomicrograph of Nissl-stained brain slice in one bird showing the typical trajectory of the electrode penetration. By carefully orienting our electrode angle, we were able to sample NCM along its entire dorsal to ventral extent. **B.** Scatter plot of noise invariance against stereotactic depth of neural recordings. Noise invariance and recording depth were significantly correlated (slope = 0.15/mm, adjusted R^2^ = 0.13, p = 0.02). The example neurons are labeled A and B on the scatter plot. **C.** Scatter plot showing the relationship between the best frequency (Y-axis) and the depth of the recording along the dorsal to ventral axis of NCM (X-axis). The solid line is the linear regression between these two variables (adjusted R2 = 0.34, p<10-3).

We found neurons with different degrees of noise invariance throughout NCM but the neurons in the ventral region tended to have highest I_cc_ (Fig. 2B). NCM also exhibits some degree of frequency tonotopy along this dimension with higher frequency tuning found in more ventral regions [18], [19]. Indeed, in our data set, we also found a strong correlation between dorsal/ventral position and the best frequency (BF) of the neuron (Fig. 2C). We estimated a neuron's best frequency from the peak of the frequency marginal of its spectral-temporal receptive field (STRF). We found a range of BF from 1300 Hz to 3300 Hz with a dorsal-ventral gradient (adjusted R^2^ = 0.34, p<10^−3^). Although the frequency range of our song stimulus and ml-noise stimulus was identical, the frequency power spectrum of song has a peak around 4 KHz [20] that could have lead to stronger and thus potentially more noise invariant responses to song for neurons with higher best frequency. A linear regression analysis between invariance and the neuron's best frequency could not confirm that hypothesis (Adjusted R^2^ = 0.06, p = 0.1). Thus, if this relationship exists, it can only have a very small effect size.

### Noise Invariance and Spectral-temporal Tuning

To further attempt to understand how noise invariance was achieved in this system, we examined how the neurons' responses for particular joint spectral-temporal patterns that are unique to song could have contributed to robust coding of song in noisy conditions. To do so we estimated the STRF of each neuron and examined the predicted response to song and to song plus noise. The STRF describes how acoustical patterns in time and frequency correlate with the neuron's response [21], [22]. The STRF can also be used as a model of the neuron to estimate predicted responses for arbitrary sound stimuli. The STRF model is often described as “linear” but can include both input and output non-linearities. In this study, the stimulus was represented as a log spectrogram and the output of the linear filter was half-wave rectified (see methods). Although we have shown that better response predictions could be obtained using additional non-linear elements such as gain control [23], in this study we have used the simpler STRF model to more explicitly describe the spectral-temporal tuning of each neuron (examples of STRF predictions are found in Fig S2).

**Figure 3:**
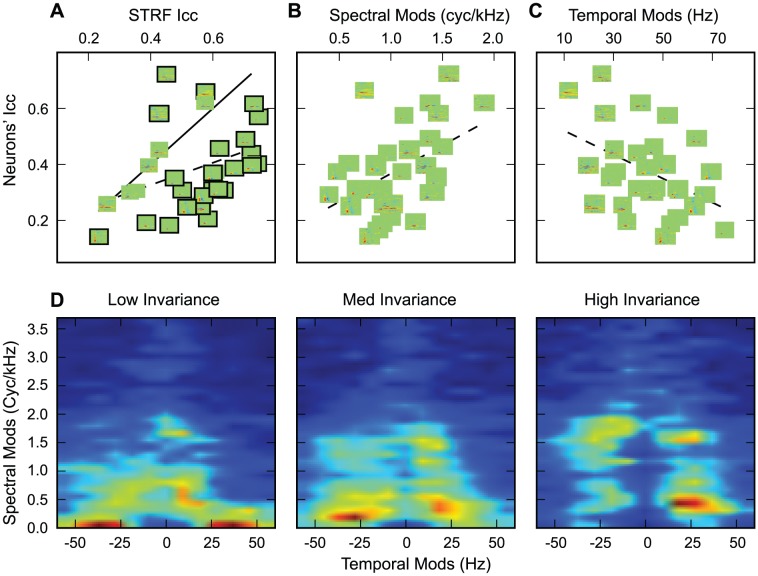
Spectral-temporal tuning and invariance. Vertical axis in A–C shows the noise invariance in the neural response. Each neuron (each point on the scatterplots) is represented by its STRF (0.25–8 kHz on the vertical axis, 0–60 ms on the horizontal). **A.** Invariance vs STRF Model Invariance. The solid line has slope 1.0, showing equal performance between the STRF model and the neural response. Neurons with significantly different performance (p<0.05, two-tailed t-test) have their receptive fields outlined. Dashed line shows regression fit (slope = 0.40, Adjusted R^2^ = 0.12, p = 0.034), indicating the positive correlation between the invariance predicted by the STRF and actual invariance. **B.** Invariance vs Spectral Modulation Tuning. Neurons sensitive to higher spectral modulations are more invariant (Adjusted R^2^ = 0.192, p = 0.007). **C.** Invariance vs Temporal Modulation Tuning. Neurons sensitive to lower temporal modulations are more invariant (Adjusted R^2^ = 0.15, p = 0.015). **D.** Ensemble modulation transfer functions for neurons grouped by invariance. Low invariance neurons (left panel, invariance<0.3, n = 11) respond to high temporal and low spectral frequency modulations. Neurons with moderate invariance (middle panel, 0.3<invariance<0.4, n = 11) transmit faster, sharper modulations. Neurons with high invariance (right panel, invariance>0.2, n = 10) respond mostly to slower and spectrally sharp sounds.

To determine whether a neuron's tuning for particular spectral-temporal features characteristic of song and less common in noise could explain the observed invariance, we use the STRF to obtain estimated responses to song and noise. We then regressed the I_CC_ values that we measured directly from the neuron's response against the I_CC_ values obtained from the predictions of STRF model (Fig. 3A). Two results come out of this analysis. First, the measured invariance and the model invariance are positively but weakly correlated showing that the neurons' STRFs can in part explain the observed noise-invariance (Adjusted R^2^ = 0.12, p = 0.034). Second, we found that, for most neurons, the degree of invariance predicted by the STRF model was greater than the one found in actual neurons. In other words, non-linearities not captured in the STRF model made these neurons less invariant. Although this result might seem surprising for an auditory region believed to be important for song recognition, it has a simple explanation. Many high-level neurons show adapting responses to sound intensity levels [24] and this common non-linear response property is not captured in this STRF model. Intensity adapting neurons would exhibit a decrease in response to the song in noise relative to the song alone due to the adaptive changes in gain. This decrease in response gain without a corresponding decrease in background rate would result in a decrease of the response's SNR.

Therefore, for the task of extracting the song from noise, the most effective non-linearities appear to be the simple thresholding non-linearity (i.e. for neurons with STRFs closest to the x = y line in Fig. 3A) or a yet to be described additional non-linearity boosts invariance (n = 3/32). Although the specific non-linearities that could be beneficial for preserving signal in noise still need to be described, previous research have characterized higher-order non-linearities response that could play an important role: neurons in NCM exhibit stimulus specific adaptation [11] and neurons in another avian secondary auditory area, CM (Caudal Mesopallium), respond preferentially to surprising stimuli [25]. These non-linearities could facilitate noise invariant responses since they tend to de-emphasize the current or expected stimulus (in this case noise like sounds) without decreasing the gain of the neuron to sound at the same frequency.

Since the STRF could partially explain the observed noise-invariance, we asked what feature of the neurons spectral-temporal tuning was important for this computation. By estimating the modulation gain from the neurons' STRFs, we found that tuning for high spectral modulations and low temporal modulations correlate with neural invariance (Fig. 3B–C). Neurons sensitive to higher spectral modulations are more invariant (Adjusted R^2^ = 0.192, p = 0.007) and neurons sensitive to lower temporal modulations are more invariant (Adjusted R^2^ = 0.15, p = 0.015). To assess the effect size of these two relationships taken together, we used multiple linear-regression with spectral and temporal modulation tuning as regressors used to explain the neurons invariance and found an adjusted R^2^ of 0.23 (p = 0.009). Thus the contributions of spectral and temporal tuning to invariance are not completely additive. The ensemble modulation transfer functions further illustrate how the spectral and temporal modulation tuning co-vary along the noise-invariance dimension (Fig. 3D). Noise invariant neurons exhibit the combination of longer integration times and sharp spectral tuning. In addition, the sharp excitatory spectral tuning was often combined with sharp inhibitory spectral tuning as well. These properties make noise-invariant neurons particularly sensitive to the longer harmonic stacks present in song (and other communication signals) even when these are embedded in noise as illustrated in the example neuron in Fig. 1 (right panel).

The generation of the observed modulation tuning properties of the more noise invariant neurons described in this study is not a trivial task: most neurons in lower auditory areas have much shorter integration times and lack the sharp excitation and inhibition along the spectral dimension that we observed here. From comprehensive surveys of tuning properties in the avian primary auditory cortex (Field L) [22], [26], we know that a small number of neurons with similar characteristics exist in these pre-synaptic areas [22]. Similarly, in the mammalian system, neurons in A1 have been shown to have a range of spectral-temporal tuning similar to that seen in birds but few with the sharp spectral tuning seen here [27], [28]. Thus it is reasonable to postulate that noise-invariance in NCM (and putatively in mammalian secondary auditory cortical regions) is the result of a series of computations that are occurring along the auditory processing stream. However, it is also known that NCM possesses a complex network of inhibitory neurons and that these play an important role in shaping spectral and temporal response properties [29]. We also found a higher concentration of noise invariant neurons in the more ventral regions of NCM but failed to find a correlation between invariance and best frequency. On the other, we found that both temporal modulation tuning (adjusted R^2^ = 0.12 p = 0.02) and spectral modulation tuning (adjusted R^2^ = 0.15 p = 0.01) were also correlated with depth: lower temporal and higher spectral modulation tuning is found in ventral regions of NCM. This organization of tuning properties is reminiscent of the organization of the primary auditory areas, field L, where the output layers have a higher concentration of neurons with longer integration times [30]. Thus both upstream and local circuitry are almost certainly involved in the creation of noise-invariant neural representations.

### Invariance and Song Selectivity

**Figure 4:**
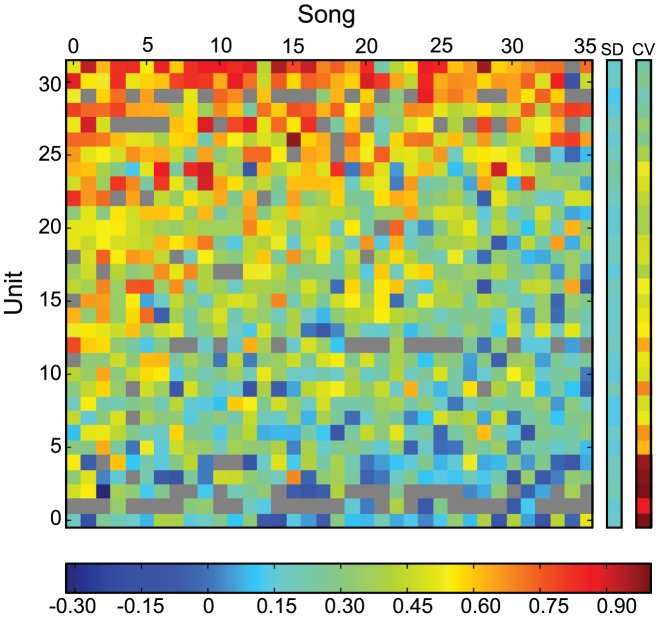
Range of invariance observed across neurons and song stimuli. Two dimensional heat plot that shows the value of the variance metric obtained for each neuron (n = 32) and each song stimuli (n = 36). The neurons are sorted from low mean invariance (bottom row) to high mean invariance (top row). The columns on the left show the standard deviation of the variance and the coefficient of variation for each neuron. The color bar is placed at the bottom of the graph and is the same for the variance, the standard deviation and the coefficient of variation. The grey cells in the matrix correspond to (neuron, stimulus) where we were not able to calculate the invariance either because of missing data or very low response rates.

Since the tuning of noise invariant neurons described by their STRF and the threshold non-linearity only describes a fraction of the invariance, we were interested in assessing whether noise invariant neurons were selective for longer sound segments such as those that might be useful to distinguish one song from another. To begin to investigate this idea, we examined the invariance of all the neurons for each song and calculated the standard deviation and the coefficient of variation (CV) of the invariance metric for each neuron. These results are shown as a two-dimensional heat map on Fig. 4. Although, the degree of invariance varied somewhat across songs (and the most invariant neurons could have invariances above 0.9 for certain sounds), the variability was remarkably low: highly invariant neurons tended to show noise-invariance to most song stimuli. The CVs for the 10 most invariant neurons were similar and all below 0.5. We therefore conclude that neurons that show a high degree of invariance could be useful to extract signal from noise not only for a specific song but also for an entire stimulus class. For example, noise invariant neurons could detect short acoustical features that are characteristic of many zebra finch songs. The STRF analysis shows that sensitivity to features up to 100 ms in duration is more than sufficient to generate in model neurons noise invariance of similar magnitude to that observed in the actual data. However, since the STRF only explains a fraction of both the observed invariance and the response, selective response properties that involve longer integration times could also be involved in the generation of noise invariant responses.

### Biologically Inspired Noise Reduction Algorithm

Inspired by our discovery of noise invariant neurons in NCM, we engineered a noise filtering algorithm based on a decomposition of the sound by an ensemble of “artificial” neurons described by realistic STRFs. We developed this algorithm both for biological and engineering purposes. Our biological goal was to demonstrate that an ensemble of noise-invariant responses such as the one observed here could indeed be used to recover a signal from noise. We also wanted to show whether an optimization process designed to extract signals from noise would rely on responses of particular artificial neurons with properties that are similar to those found in the biology. Finally, we also wanted to explore to what extent is the invariance of signal in noise dependent on the exact statistics of the signal and noise stimuli. Our engineering goal was to develop a real-time algorithm inspired by the biology that could potentially be used in clinical applications such as hearing aids and cochlear implants or in commercial applications involving automatic speech recognition [31]. In hearing aids, various forms of noise reduction have been shown to offer an incremental improvement in the listening experience [32], [33] though listening to speech in noisy environments remains the principal complaint of hearing aid users [34]. In addition, none of the current noise reduction algorithms have led to improvements in speech intelligibility [35], [36].

Our ensemble of artificial neurons can be thought of as a modulation filter bank because the response of each neuron quantifies the presence and absence of particular spectral-temporal patterns as observed in a spectrogram and, contrary to a frequency filter bank, not solely the presence or absence of energy at a particular frequency band. In other words, the STRFs can be thought of as “higher-level” sound filters: if lower-level sound filters operate in the frequency domain (for example removing low frequency noise such as the hum of airplane engines), these high-level filters operate in the spectral-temporal modulation domain. In this joint modulation domain, sounds that have structure in time (such as beats) or structure in frequency (such as in a musical note composed of a fundamental tone and its harmonically related overtones) are characterized by specific temporal and spectral modulations. A spectral-temporal modulation filter could then be used to detect sounds that contain particular time-frequency patterns while filtering out other sounds that might have similar frequency content but lack this spectral-temporal structure. Similar decompositions have also been proposed and used by others for the efficient processing of speech and other complex signals [37], [38], [39].

Noise filtering with such a modulation filter bank can be described as series of signal processing steps: i) decompose the signal into frequency channels using a frequency filter bank; ii) represent the sound as the envelope in each of the frequency channels, as it is done in a spectrogram; iii) filter this time-frequency amplitude representation by a modulation filter bank to effectively obtain a filtered spectrogram; iv) invert this filtered spectrogram to recover the desired signal. Although each of these steps involves relatively simple signal processing, two significant issues remain. First, one has to choose the appropriate gain on the modulation filters in order to detect behaviorally relevant signals over noise. Second, the spectrogram inversion step requires a computationally intensive iterative procedure [40] that would prevent such a modulation filtering procedure to operate in real time or with minimal delays. Our algorithm solves these two issues. We have eliminated the spectrographic inversion step and instead use the output of the modulation filter bank to generate a time-varying gain vector that can directly operate on the output of the initial frequency filter bank. Second, we propose to find optimal fixed gains on the modulation filter bank by minimizing the error between a desired signal and the output of the filtering process in the time domain. Then once the modulation filter weights are fixed, the algorithm can operate in real-time with a delay that is only dependent on the width of the STRF in the modulation filter bank.

**Figure 5:**
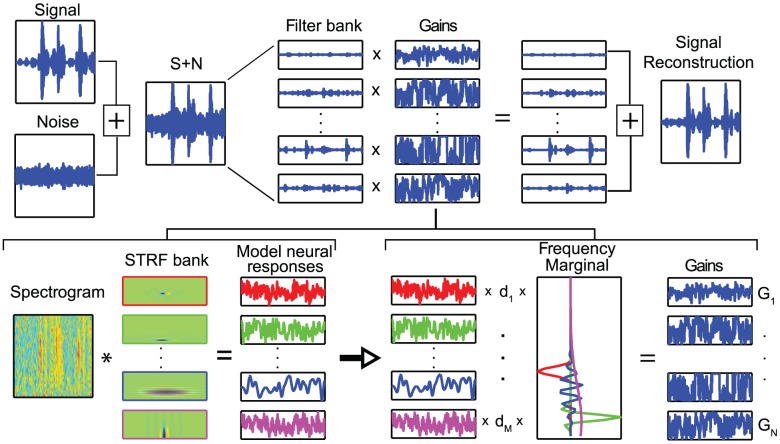
Noise reduction algorithm. We implemented a biologically inspired noise-filtering algorithm using an analysis/synthesis paradigm (top row) where the synthesis step is based on a STRF filter bank decomposition. The bottom row shows the model neural responses obtained from a sound (spectrogram of noise-corrupted song) using the filter bank of biologically realistic STRFs. These responses are then weighed optimally with weights d_1_,..,d_M_ to select the combination of responses that are most noise-invariant. The weighted responses are then transformed into frequency space by multiplying the weighted responses by the frequency marginal of the corresponding STRF (color-matched on the figure) to obtain gains as a function of frequency. The top row illustrates how these time-varying frequency gains can then be applied to a decomposition of the sound into frequency channels allowing for the synthesis step and an estimate of the clean signal. This technology is available for licensing via UC Berkeley's Office of Technology Licensing (Technology: Modulation-Domain Speech Filtering For Noise Reduction; Tech ID: 22197; Lead Case: 2012-034-0).

The various steps in our algorithm are illustrated on Fig. 5. Both the analysis and synthesis steps of the algorithm use a complete (amplitude and phase) time-frequency decomposition of the sound stimuli. This time-frequency decomposition is obtained from a frequency filter bank of N-linearly spaced band-pass filter Gaussian shaped channels located between 250 Hz and 8 kHz. The amplitude of these N narrow-band signals is obtained using the Hilbert transform (or rectification and low-pass filtering) to generate a spectrogram of the sound. This spectrographic transformation is identical to the one that we use for the estimation of the STRFs (see methods).

The analysis step in the algorithm involves generating an additional representation of the sounds based on an ensemble of model neurons fully characterized by their STRF. These STRFs are designed to efficiently encode the structure of the signal and the noise, allowing them to be useful indicators of the time-course of signal in a noisy sound. For this study, we used a bank of STRFs that were designed to model the STRFs found throughout the auditory pallium, including STRFs not only from neurons in NCM but also the field L complex [22]. The log spectrogram of the stimulus is convolved with each STRF to obtain model neural responses: 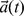 of dimension M. The crux of our algorithm is to transform these neural responses back into a set of time varying frequency gains, 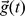 of dimension N. These frequency gains will then be applied to the corresponding frequency slices in the time-frequency decomposition of the sound to synthesize the processed signal. 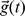 is a function of the sum of all model neural responses each scaled by an importance weighting, 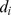, and then multiplied by the frequency marginal of the corresponding neuron's STRF:
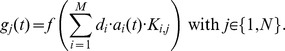
The function f was chosen to be the logistic function in order to restrict the gains to lie between a lower bound, representing maximal attenuation, and 0 dB, representing no attenuation. K_i,j_ is the frequency marginal value of neuron i for the frequency band centered at j, and it was obtained from the frequency marginal of each STRF. Using these gains, we then synthesized a processed signal:
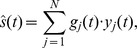
where 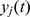 is the narrow-band signal from the frequency filter j obtained in the time-frequency decomposition of the song + noise stimulus, 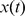. The optimal set of weights, 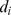, was learned by minimizing the squared error 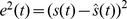 through gradient descent.

To assess the quality of our algorithm, we compared it to 3 other noise reduction schemes: the optimal classical frequency Wiener filter for stationary Gaussian signals (OWF), a state-of-the-art spectral subtraction algorithm (SINR) used by a hearing aid company, and the upper bound obtained by an ideal binary mask (IBM). The optimal Wiener filter is a frequency filter whose static gain depends solely on the ratio of the power spectrum of the signal and signal + noise. The state-of-the-art spectral subtraction algorithm uses a time variable gain just as in our algorithm but based on a running estimate of noise and signal spectrum. This algorithm was patented by Sonic Innovations (US Patent 6,757,395 B1) and is currently used in hearing aids. The IBM procedure used a zero-one mask applied to the sounds in the spectrogram domain. The mask is adapted to specific signals by setting an amplitude threshold. Ideal binary masks require prior knowledge of the desired signal and thus can be considered as an approximate upper bound on the potential performance of general noise reduction algorithms [41].

**Figure 6:**
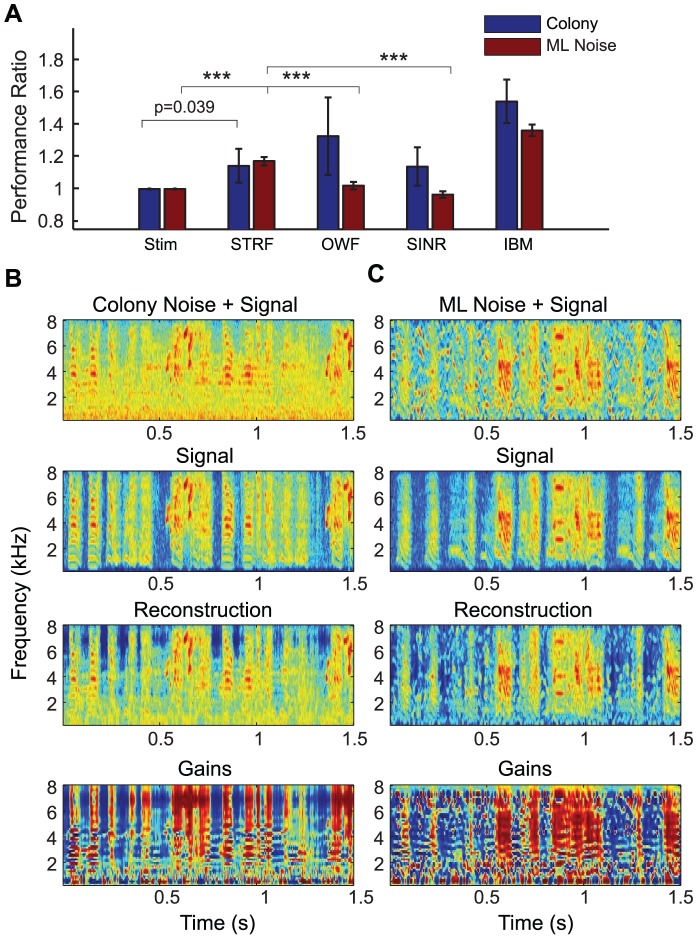
Performance of STRF based noise-reduction. **A.** Performance of three noise reduction algorithms (STRF, OWF, SINR) and lower and upper bounds (Stim, IBM) on song embedded in colony noise or modulation-limited (ML) noise. The performance ratio (y-axis) depicts the improvement in noise levels over the noise-corrupted signal, as measured by the cross-correlation in the log spectrogram domain, with the error bars representing one standard deviation across five noisy stimuli. On the x-axis are the models we have tested, where “Stim” is the noise-corrupted signal, “STRF” is the model presented here, “OWF” is the optimal Wiener filter, “SINR” is a spectral subtraction algorithm similar to STRF but based on engineering constructs, and “IBM” is an ideal binary mask. **B.** Spectrograms of the signal masked with noise from the zebra finch colony, the clean zebra finch song, and our signal reconstruction, followed by the time-frequency gains. **C.** Same as B but for modulation-limited noise. Sounds can be found in the SI.

As shown on Fig. 6A, with relatively little customization and exploration (for example in the choice of the set of artificial STRFs) our algorithm performed strikingly well: our algorithm performed significantly better than both the classical frequency Wiener filter and the SINR algorithm for a song embedded in ml-noise and similarly to the SINR algorithm for a song embedded in colony noise. The quality of the noise filtering can also be assessed by examining the time-varying gains shown on bottom row in Figs 6B and C: without any *a priori* knowledge of the location of the signal in time (and contrary to the IBM), the time-varying gains can pick out when the signal occurs in the noise. Moreover, the gains are not constant for all frequencies but instead are also able to pick out harmonic structure in the sound. The quality of the reconstruction can also be visually assessed by examining the spectrograms shown in that figure or listening to the demos provided as supplemental material.

**Figure 7:**
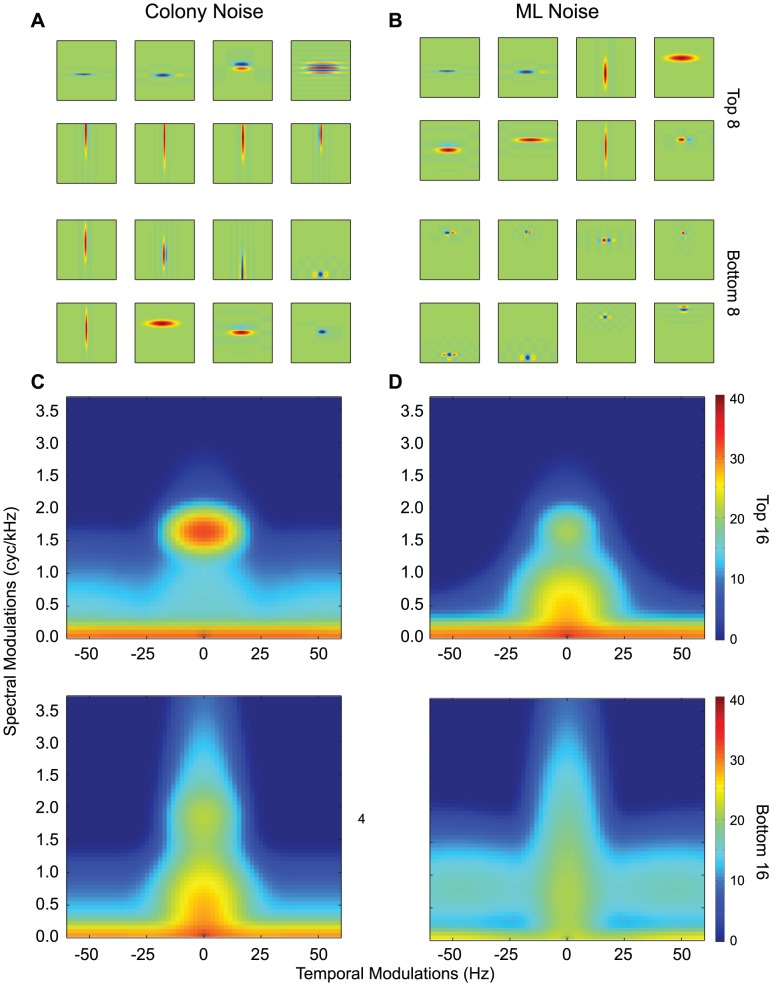
Model STRFs for noise reduction. **A**. The eight most positively (top) and most negatively (bottom) weighted STRFs from the noise reduction algorithm trained with a background of colony noise. **B**, Same as in A, but for the model trained with a background of modulation-limited noise. **C.** The ensemble modulation transfer functions for the top 16 and bottom 16 STRFs for the model trained in colony noise, sorted as in A. **D** Same as in C, but for the model trained in modulation-limited noise.

We are now able to answer our questions. First, as quantified above, using an ensemble of physiologically realistic noise-invariant responses, we show that one is able to recover the distorted signal with remarkable accuracy. Second, we were also able to compare the properties of the STRFs in the model that had the biggest importance gains (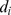) with those found in noise-invariant neurons in NCM. As shown on Fig. 7A &B, these STRFs are composed both of narrow band neurons with long integration times as observed in our data set and also broad band neurons with very short integration time. The eMTF shown in Fig. 7C&D further quantify these results. Thus, the noise invariant neurons found in NCM are well represented in by the model STRFs tuned for high spectral modulation and low temporal modulations. NCM also has neurons tuned to faster temporal modulations but the majority of these neurons had narrow band frequency tuning (or high spectral modulations) and these neurons are therefore not particularly effective at rejecting noise stimuli. Fast broad-band neurons are however found in the avian primary auditory forebrain [22], [26] and could thus play a role, as part of an ensemble, in the signal and noise separation. Our third question regarded the sensitivity of noise-invariant neurons to the particular choice of signal and noise. The modeling shows that the importance weights obtained for filtering out ml-noise were slightly different that the weights obtained for filtering colony noise. This relatively small effect can be visually assessed by comparing the highest weighted STRFs for each noise class shown in Fig. 7A versus 7B. These results suggest that slightly different sets of invariant-neurons depending on the statistical nature of the signal and noise but that these effects might be rather small. In addition, we found no correlation between the magnitude of importance weights of the artificial neurons and their BF. Thus, we also predict that the modulation tuning properties of noise-invariant neurons that we described here would apply to a relatively large relevant set of natural signals and noise. This is in part possible because many forms of environmental noise, including noise resulting from the summation of multiple sound signals, have similar modulation structure characterized by a concentration of energy at very low spectral modulations and low to intermediate temporal modulations. In converse, communication signals can have significant energy in regions combining either high spectral modulations with low temporal modulations or high temporal modulations with low spectral modulations [17] [42].

Both in the model and in the biological system, given a complete modulation filter bank, the importance weights for a given signal and noise could be learned quickly through supervised learning. Moreover, after learning, the algorithm can easily be implemented in real-time with minimal delay. Thus, the algorithm is particularly useful with adaptive weights or if the statistics of the noise and signal are known, both of which are true in the biological system. Finally given its performance and the advantages described above, we also believe that this noise filtering approach could be useful in clinical applications, such as hearing aids or cochlear implants, or in consumer applications such as noise canceling preprocessing for automatic speech recognition.

In summary, we have shown the presence of noise-invariant neurons in a secondary auditory cortical area. We show that a fraction of the noise-rejecting property can be explained by the spectral-temporal tuning of the neurons. However, tuning properties that are not well captured by the STRF can also both increase or decrease noise-invariance and these properties will have to be examined in future work. We have also described a novel noise reduction algorithm that uses a modulation filter-bank akin to the STRFs found in the avian auditory system. The performance of this algorithm in noise reduction was excellent and similar or better than the current state-of-the-art algorithms used in hearing aids. The model also illustrates some fundamental principles and allowed us to make stronger statements on the scope of our biological findings. The fundamental principles are, first, that signal and noises can have a distinct signature in the modulation space while overlapping in the frequency space and that therefore filtering in this domain can be advantageous. Second, that although modulation filtering is a linear operation in the spectrogram domain, that both the generation of a spectrogram and the re-synthesis of a clean signal require non-linear computations. We argue that the spectral-temporal properties that are found in higher auditory areas and that are particularly efficient at distinguishing noise modulations from signal modulations are the result of a series of non-linear computations that occurred in the ascending auditory processing stream. The model also shows that a real-time re-synthesis of a cleaned signal could be obtained with additional non-linear operations or, in other words, that a real-time spectrographic inversion is possible. Finally, our modeling efforts show that the noise-invariant findings described here for a song as a chosen prototypical signal and a modulation-limited noise as the chosen prototypical noise would also apply to other signals and noise. However, the involvement of neurons with slightly different tuning or adaptive properties would be needed to obtain optimal signal detection. Given the behavioral experiments that have shown that birds excel at auditory scene analysis tasks both in the wild [9] and in the lab [43], [44] and given our increasing understating of the underlying neural mechanisms [45], the birdsong model shows great promise to tackle one of the most difficult and fascinating problems in auditory sciences: the analysis of a sound scape into distinct sound objects.

## Methods

### Neurophysiology and Histology

All animal procedures were approved by our institutional Animal Care and Use Committee. Neurophysiological recordings were performed in four, urethane anesthetized adult zebra finches to obtain 50 single unit recordings in areas NCM and potentially field L (see below). We used similar neurophysiological and histological methods to characterize other regions of the avian auditory processing stream and detailed descriptions can be found there [22]. The methods are summarized here and differences when they exist are noted.

To obtain recordings from NCM, we used more medial coordinates than our previous experiments. With the bird's beak fixed at a 55° angle to the vertical, electrodes were inserted roughly 1.2 mm rostral and 0.5 mm lateral to the Y-sinus. We made extracellular recordings from tungsten-parylene electrodes having impedance between 1 and 3 MΩ (A-M Systems). Electrodes were advanced in 0.5 µm steps with a microdrive (Newport), and extracellular voltages were recorded with a system from Tucker-Davis Technologies (TDT).

In all cases, the extracellular voltages were thresholded to collect candidate spikes. Each time the voltage crossed the threshold, the timestamp was saved along with a high-resolution waveform of the voltage around that time (0.29 ms before and 0.86 ms after for a total of 1.15 ms). After the experiment, these waveforms were sorted using SpikePak (TDT) to assess unit quality. We sorted spike waveforms using a combination of PCA and waveform features (maximum and minimum voltage, maximum slope, area). We assessed clustering qualitatively and verified afterwards that the resulting units had Inter-Spike-Interval distributions where no more than 0.5% of the intervals were less than 1.5 ms.

In each bird, we advanced the electrode in 50 µm steps until we found auditory responses. At that point we recorded activity in 100 µm steps. When we no longer found auditory responses, we moved the electrode 300 µm further, made an electrolytic lesion (2 uA×10 s), advanced another 300 µm, and made a second identical lesion. These lesions were used to find the electrode track post-mortem and to calibrate the depth measurements.

At the end of the recording session, the bird was euthanized with an overdose of Equithesin and transcardially perfused with 0.9% saline, followed by 3.7% formalin in 0.025 M phosphate buffer. The skullcap was removed and the brain was post-fixed in 30% sucrose and 3.7% formalin to prepare it for histological procedures. The brain was sliced parasagittally in 40 µm thick sections using a freezing microtome. Alternating brain sections were stained with both cresyl violet and silver stain, which were then used to visualize electrode tracks, electrolytic lesions and brain regions.

All of our electrode tracks sampled NCM from dorsal to ventral regions. Some of the more dorsal recordings (shallower depths) could have been in subregions L or L2b of the Field L complex as the boundary between either of these two regions and NCM proper is difficult to establish [46], [47]. It is possible therefore that the correlation between degree of invariance and depth also reflects lower invariance observed in the field L complex and higher invariance in NCM proper.

### Sound Stimuli

Stimuli consisted of zebra-finch songs, roughly 1.6–2.6 seconds in length, recorded from 40 unfamiliar adult male zebra finches played either in isolation or in combination with a background of synthetic noise (song+ml-noise stimuli in main text).

The masking noise in the neurophysiological experiments was synthetic and obtained by low-pass filtering white noise in the modulation domain following the procedure described in [48]. This modulation low-pass filter had cutoff frequencies of ω_f_ = 1.0 cycles/kHz and ω_t_ = 50 Hz and gain roll off of 10 dB/(cycle/kHz) and 10 dB/10 Hz. The cutoff modulation frequencies were chosen in order to generate noisy sounds with similar range of modulation frequencies found in environmental noise [17]. In addition, most of the modulations found in zebra finch song are well masked by this synthetic noise although it should be noted that song also includes sounds features with high spectral modulation frequencies (above 2 cycles/kHz) and high temporal modulation frequencies (above 60 Hz). The frequency spectrum of the ml-noise was flat from 250 Hz to 8 kHz completely overlapping the entire range of the band-passed filtered songs we used in the experiments. Thus, although, different results could be found with noise stimuli with different statistics, we carefully designed our masking noise stimulus to both capture the modulation found in natural environmental noise while at the same time completely overlapping the frequency spectrum of our signal. The frequency power spectrum of these signals can be found in [20].

We have also shown that such ml-noise is an effective stimuli for midbrain and cortical avian auditory neurons in a sense that it drives neuron with high response rates and high information rates [20]. ML-noise is also very similar to the dynamic noise ripples described in [49] and used in many neurophysiological studies to characterize high-level mammalian auditory neurons. We also recorded responses to the ml-noise masker alone but these data were not analyzed for this study.

All song and ml-noise stimuli were processed to be band limited between 250 Hz and 8 kHz and to have equal loudness using custom code in Matlab. The sounds were presented using software and electronics from TDT. Stimuli were played over a speaker at 72 dB C-weighted average SPL in a double-walled anechoic chamber (Acoustic Systems). The bird was positioned 20 cm in front of the speaker for free-field binaural stimulation.

Each of the combined stimuli consisted of a different ml-noise sound sample, randomly paired with one of the songs. The noise stimulus began five to seven seconds after the previous stimulus, and the song began after a random delay of 0.5 to 1.5 seconds after the onset of the noise. Thus for each trial the same song is paired with a different noise sample and at a different delay. In the combined presentations, the noise stimuli were attenuated by 3 dB to obtain a signal to noise ratio (SNR) of 3 dB.

We played four trials at each recording location, each consisting of a randomized sequence of 40 songs, 40 masking noise stimuli, and 40 combined stimuli. Stimuli were separated by a period of silence with a length uniformly and randomly distributed between five and seven seconds.

### Neural Data Analysis

We used custom code written in MATLAB, Python and R for all of our analyses.

We assessed responsiveness using an average z-score metric for each stimulus class. The z-score is calculated as follows:
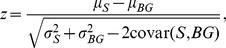
where μ_S_ is the mean response during the stimulus, μ_BG_ is the mean response during the background, σ_S_^2^ is the variance of the response during the stimulus, and σ_BG_^2^ the variance of the response during baseline. The background rates were calculated using the 500 ms periods preceding and following each stimulus. Using a cutoff of z≥1.5 for either ml-noise or song stimuli, 32 of the 50 single units were determined to be responsive.

To measure invariance, we evaluated the similarity between the responses to song and song + ml-noise by computing two measures: 1) the correlation coefficient between the PSTH for each corresponding response and 2) the ratio of the SNR in the neural response to song+noise and the SNR in the response to song alone.

If the PSTH for song is called 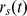 and the PSTH obtained in response to song+noise is called 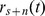, then the correlation coefficient is given by:
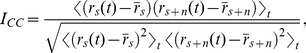
where the <> are averages across time samples. We called this correlation coefficient, the correlation invariance or the invariance for short. The correlation coefficient is bounded between −1 and 1 and measures the linear similarity in the response after mean subtracted and scaling. Thus a response to song+noise with a deviation from its mean rate that is similar in shape but much smaller than the time-varying response to song alone will have a very high CC invariance. A better measure of invariance might therefore take into account both the mean PSTH rate as a proxy for noise and the deviations from this rate as a measure of signal. Thus, for the response to song alone, we define the signal power as 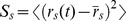 and the noise power as 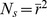 for a signal to noise ratio of:
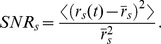
For the response to the song+noise, we wanted to determine the fraction of the time varying-response that was related to the song. For that purpose, we used 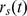 as a regressor to obtain an estimate of 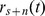:
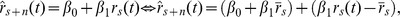
where 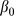 and 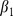 are the coefficients obtained from the normal solution for linear regression.

The signal to noise ratio for the response to song+noise is then:
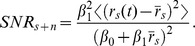
And the SNR invariance is given by the ratio of the two SNRs:
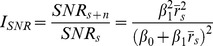
As shown on Fig S1A, the two metrics ended up being highly correlated: the correlation coefficient between 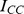 and the log of 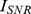 is r = 0.94 (p<10^−6^) and we decided to use I_CC_ in the main text. However, the calculation of 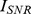 also provides useful information in terms of the absolute magnitude of the invariance. For example, it shows that the SNR in the response for the seven most invariant cells is decreased by 5 to 10 dB when the song in presented in noise. Thus, even for these noise-robust neurons the loss of signal quality is present. Similarly, one can examine the value of the linear regression coefficient, 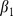 on Fig S1B. This coefficient is always less than one showing that the responses to the song signal in the song+noise stimulus is always reduced. 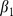 is also highly correlated with 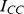 but always smaller. Together this shows that although the shape of the time-varying response is often very well preserved in noise-invariant neurons, that the magnitude of this response is decreased resulting in significant losses in signal power (informative time-varying firing rate) relative to noise power (mean firing rate).

In the calculations above, the PSTH was obtained by smoothing spike arrival times using a 31 ms Hanning window. The bias introduced by the small number of trials used to compute each PSTH was correcting by jackknifing. The single-stimulus results indicate a small but consistent negative bias in the four-trial estimates. We then computed the invariance as the mean of the individual bias-corrected correlations obtained for each 40 stimulus.

For each responsive single unit, we estimated the neuron's STRF from their responses to song alone. The STRF were obtained using the *strfLab* neural data analysis suite developed in our laboratory (strflab.berkeley.edu). The STRFs were estimated by regularized linear regression. The algorithm is implemented as a Ridge Regression in *strfLab* (*directfit* training option). Because of the 1/f^2^ statistics of song, the ridge regression hyper parameter acts as a smoothing factor on the STRF. In addition, we used a sparseness hyper-parameter that controls the number of non-zero coefficients in the STRF. Optimal values of the two hyperparameters were found by Jackknife cross-validation (see [21], [50] for more details). The stimulus representation used for the STRF was the log of the amplitude of the spectrogram of the sound obtained with a Gaussian shaped filter bank of 125 Hz wide frequency bands. Time delays of up to 100 ms were used to assess the cross-correlation between the stimulus and the response. Performance of the estimated final best STRF was then quantified with a separate validation data set.

We assessed the performance of each STRF using coherence and the normal mutual information as described in [51], [52]. First, we compute the expected coherence between two single response trials; we then compute the coherence between the STRF prediction and the average response. The coherence is a function of frequency between zero and 1 that measures the correlation of two signals at each frequency. To obtain a single measure of correlation, one can compute the normal mutual information (MI). We then computed the normal MI for the two coherences, calling the first the “response information” and the second the “predicted information”. The ratio of the predicted information to the response information is the performance ratio, and provides a measure of model performance that is independent of the variability of the neuron [52]. In all of our receptive field analyses, we used only STRFs that predict sufficiently well, defined here as having predicted information of at least 1.2 bits/second and a performance ratio of at least 20%. The STRF performance was not correlated with either the responsiveness of the neuron, as measured by their z-score, or the degree of invariance (data not shown).

To further examine the gain of the neuronal response as a function of temporal and spectral modulations, we also represented each STRF in terms of its Modulation Transfer Function (MTF). The MTF is obtained by taking the amplitude of 2 dimensional Fourier Transform of the STRF [42]. For each neuron, we also computed the center of mass of its MTF to estimate its best spectral and temporal modulation frequencies

To calculate the invariance metrics for the STRF model, we first obtained the predicted response to the song+ml-noise stimulus for each trial. Using these in place of the actual responses, we then computed an invariance metrics for the STRF model by comparing the predicted responses to the actual response obtained for song alone. In this manner, we were able to directly compare the STRF model invariance with the invariance calculated for the actual neuron. We used a two-tailed t-test to compare the distribution of similarity values for the 40, four-trial linear predictions to the 40 actual four-trial responses.

Fig. S2 illustrates the methodology and shows the STRF, MTF, neural responses and predictions to both song and song+ml-noise for two additional example neurons: one with relatively low noise-invariance and one with relatively high noise-invariance.

### Noise Filtering Algorithm Using the Modulation Filter Bank Model

Following directly from the premise that neurons in area NCM selectively respond to spectral-temporal modulations present in zebra finch songs, even in the presence of corrupting background noise, we developed a noise reduction scheme that would exploit this property. Our algorithm falls in the general class of single microphone noise reduction (SMNR) algorithms using spectral subtraction. The core idea in spectral subtraction is to estimate the frequency components of the signal from the short time Fourier components of the corrupted signal. The estimated signal frequency components are obtained by multiplying the Fourier components of signal+noise by a gain function. This is the synthesis part of the algorithm. The gain function can vary both in frequency and time. The form and estimation of the *optimal* gain function is the analysis step of the algorithm and its design is the principal focus of the novel development of the state-of-the art SMNR algorithms.

Both the analysis and synthesis step in our algorithm used a complete (amplitude and phase) time-frequency decomposition of the sound stimuli (Fig. 5). This time-frequency decomposition was obtained from a frequency filter bank of N-linearly band-pass filter Gaussian shaped channels located between 250 Hz and 8 kHz (BW = 125 Hz). N was set at 60 for all simulations. The amplitude of these N narrow-band signals could then be obtained using the Hilbert transform to generate a spectrogram of the sound.

The analysis step in the algorithm involved generating an additional representation of the sounds based on an ensemble of M model neurons fully characterized by their STRF. The model STRFs were parameterized as the product of two Gabor functions describing the temporal and spectral response of the neuron:
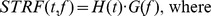


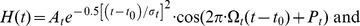


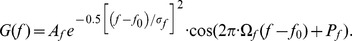



The parameters of these Gabor functions (e.g. for time: 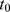, the temporal latency; 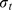, the temporal bandwidth; 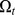, the best temporal modulation frequency; and 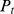, the temporal phase) were randomly chosen using a uniform distribution over the range of those found in area NCM (present study) and Field L [22]. The number of model neurons, M, was not found to be critical as long as the population of STRFs sufficiently tiled the relevant modulation space. M was set to be 140 for the results shown. To obtain the representation of sounds in this “neural space”, the log spectrogram of the stimuli was convolved by each STRF to obtain the model neural response: 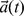 of dimension M. As explained in the main text, we then used these activation functions to obtain a set of optimal time varying frequency gains, 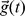 of dimension N. These frequency gains are then be applied to the corresponding frequency slices in the time-frequency decomposition of the sound to synthesize the processed signal using:
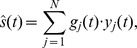
where 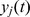 is the narrow-band signal from the frequency filter j obtained in the time-frequency decomposition of the song + noise stimulus, 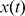.

The optimal set of weights, 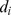, needed to obtain the optimal gains, 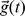 (see Results) was learned by minimizing the squared error 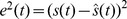 through gradient descent. For this purpose, training stimuli were generated by summing together a 1.5 s song clip and a randomly selected chunk of either ml-noise or zebra finch colony noise of the same duration. To match the experimental results, both the song, 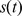, and the noise, 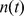, were first high-pass filtered above 250 Hz and low-pass filtered below 8 kHz, and then resampled to a sampling rate of 16 kHz. The song and noise were weighted to obtain a SNR of 3 dB, although similar results were found with lower SNR's.

Training was performed on all instances of the signal + noise samples. Weights were determined by averaging across values obtained through jack-knifing across this data set ten times with 10% of the data held out as an early stopping set. Noise reduction was then validated and quantified on a novel song in novel noise. Examples of noise corrupted signals and filtered signals that correspond to the spectrograms shown in Fig. 6 can be found in the supplemental online material: *Audio S1*, Zebra finch song masked by ml-noise; *Audio S2*, the recovered song signal; *Audio S3*, the original song signal; *Audio S4*, Zebra finch song masked by colony noise; *Audio S5*, the recovered song signal; *Audio S6*, the original song signal.

To assess the performance of our model, we computed the cross-correlation between the estimate and the clean signal in the log spectrogram domain. We then took the ratio of this cross-correlation and the value obtained prior to attempting to de-noise the stimulus to obtain a performance ratio. As summarized in the text, we then compared our algorithm to other noise reduction schemes. For this purpose, we also estimated the performance ratio for three other spectral subtraction noise algorithms: the optimal Wiener filter (OWF), a variable gain algorithm patented by Sonic Innovations (SINR) and the ideal binary mask (IBM). The optimal Wiener filter is a frequency filter whose static gain depends solely of the ratio of the power spectrum of the signal and signal + noise. In our implementation, the Wiener filter was constructed using the frequency power spectrum of signal and noise from the training set and then applied to a stimulus from the testing set (of the same class). The spectral subtraction algorithm for Sonic Innovations used a time variable gain just as in our implementation. Also, as in our implementation, the analysis step for estimating this gain was based on the log of the amplitude of the Fourier components. However, the gain function itself was estimated not from a modulation filter bank but estimating the statistical properties of the envelope of the signal and noise in each frequency band (US Patent 6,757,395 B1). We used a Matlab implementation of the SINR algorithm provided to us by Dr. William Woods of Starkey Hearing Research Center, Berkeley, CA. Optimal parameters for the level of noise reduction and the estimation of the noise envelope for that algorithm were also obtained on the training signal and noise stimuli and the performance was cross-validated with the test stimuli. The IBM procedure used a zero-one mask applied to the sounds in the spectrogram domain. The mask is adapted to specific signals by setting an amplitude threshold. Binary masks require prior knowledge of the desired signal and thus should be seen as an approximate upper bound on the potential performance of general noise reduction algorithms. Although these simulations are far from comprehensive, they allowed us to compare our algorithm to optimal classical approaches for Gaussian distributed signals (OWF), to a very recent state-of-the-art algorithm (SINR) and to an upper bound (IBM). For commercial applications, our noise-reduction algorithm is available for licensing via UC Berkeley's Office of Technology Licensing (Technology: Modulation-Domain Speech Filtering For Noise Reduction; Tech ID: 22197; Lead Case: 2012-034-0).

## Supporting Information

Audio S1 **The Zebra finch song from Audio S3 masked by modulation limited-noise.**
(WAV)

Audio S2 **The recovered song signal after the corrupted signal in Audio S1 was processed by our biological inspired noise-reduction algorithm.**
(WAV)

Audio S3 **An example of a Zebra finch song labeled as a signal since it will be masked (in Audio S1) and recovered (in Audio S2).**
(WAV)

Audio S4 **The Zebra finch song from Audio S6 masked by colony noise.**
(WAV)

Audio S5 **The recovered song signal after the corrupted signal in Audio S4 was processed by our biological inspired noise-reduction algorithm.**
(WAV)

Audio S6 **An example of a Zebra finch song labeled as a signal since it will be masked (in Audio S4) and recovered (in Audio S3).**
(WAV)

Figure S1
**Comparison of the correlation invariance (I_CC_) and the SNR invariance (I_SNR_).** **A.** Scatter plot showing the strong correlation between the I_SNR_ (in dB units) and I_CC_: r = 0.96, p<10^−6^. **B.** Scatter plot between the non-normalized linear regression coefficient, 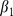, and the normalized measure of invariance, I_CC_. These two measures are also highly correlated: r = 0.96, p<10^−6^.(EPS)

Figure S2 **Example of STRF and STRF predictions for two cells.** **Column A**: A low noise-invariant cell (invariance = 0.25), Cell 1 and **Column B**: a high noise-invariant cell (invariance = 0.65), Cell 2. Top row shows the STRF and the corresponding MTF. Second row shows the spectrogram of one song stimulus. Third and fourth row show the neural responses as a spike raster (top) and a PSTH (below) to the song presented alone. In the PSTH plot, the actual neural response is in blue and the prediction obtained from the STRF is in red. Spike raster for response of low noise-invariant cell to masked song. The fifth and sixth row show the responses to the song presented over a masker of ml-noise.
(EPS)
